# Using the DASH Questionnaire to Evaluate Donor Site Morbidity of the Serratus Anterior Free Flap in Head and Neck Reconstruction: A Multicenter Study †

**DOI:** 10.3390/jcm11092397

**Published:** 2022-04-25

**Authors:** Stefan Janik, Julian Pyka, Muhammad Faisal, Stefan Grasl, Pawel Golusinski, Blažen Marijić, Rudolf Seemann, Boban M. Erovic

**Affiliations:** 1 Department of Otorhinolaryngology, Head and Neck Surgery, Medical University of Vienna, 1090 Vienna, Austria; stefan.janik@meduniwien.ac.at (S.J.); stefan.grasl@meduniwien.ac.at (S.G.); 2 Department of Oral and Maxillofacial Surgery, Medical University of Vienna, 1090 Vienna, Austria; julian.pyka@meduniwien.ac.at; 3 Department of Surgical Oncology, Shaukat Khanum Memorial Cancer Hospital, Lahore 54000, Pakistan; maxfas@live.com; 4 Department of Otolaryngology and Maxillofacial Surgery, University of Zielona Gora, 65-046 Zielona Gora, Poland; p.golusinski@cm.uz.zgora.pl; 5 Institute of Head and Neck Diseases, Evangelical Hospital, 1180 Vienna, Austria; blazen.marijic@uniri.hr (B.M.); r.seemann@ekhwien.at (R.S.); 6 Department of Otorhinolaryngology, Head and Neck Surgery, Clinical Hospital Center Rijeka, Faculty of Medicine, University of Rijeka, 51000 Rijeka, Croatia

**Keywords:** serratus anterior free flap, DASH score, donor site morbidity, head and neck reconstruction

## Abstract

**Objective:** To evaluate donor site morbidity of the serratus anterior free flap (SAFF) in head and neck reconstruction. **Methods:** The Disabilities of the Arm, Shoulder and Hand (DASH) questionnaire (0 no disability to 100 most severe disability) was applied to 20 patients (M: 16; F: 4) who underwent ablative surgery and reconstruction of the head and neck using a SAFF. Applications, as well as the donor site, recipient site and flap-related complications, were evaluated. **Results:** SAFF was mainly used for tongue (*n* = 11; 55.0%) and pharyngeal reconstruction after a laryngopharyngectomy (*n* = 4; 20.0%). The majority of patients presented with stage IV disease (*n* = 12; 60%) and had undergone previous radiotherapy (*n* = 14; 70%). Our free flap survival rate was 88.9% and the pectoralis major muscle flap (PMMF) was used in 5 patients as a salvage option to reconstruct pharyngeal defects. The mean/median DASH score was 21.6/19.9 (healthy norm 10.1), indicating only mild to moderate disability. However, free flap failure and the additional harvest of PMMF multiplies donor site morbidity since it was associated with a 3- and 2.6-times higher DASH score (46.0 vs. 15.5; *p* = 0.039 and 39.9 vs. 15.47; *p* = 0.081). **Conclusions:** The SAFF represents a versatile flap for head and neck reconstruction with low donor site morbidity.

## 1. Introduction

The serratus anterior free flap (SAFF), first described in 1982, is based on the thin, flat and finger-shaped serratus anterior muscle originating from the lateral scapula and inserting anteriorly on the first nine ribs [1,2]. The most inferior digitations, which are typically used for reconstructions, are supplied by the serratus branch of the thoracodorsal artery that pierces into the subscapular artery in turn. Thereby, the SAFF could be used alone or as a composite flap based on the subscapular system either. If necessary, up to five digitations can be harvested off the same pedicle and slips can be even further separated to gain up to 10 sub-slips with different muscle vectors [3,4,5].

Due to its thinness, pliability, ease of harvest and reliable vascular supply, the SAFF has become an important reconstructive option for numerous defects. Since the SAFF can be harvested as a myocutaneous or osteomyocutaneous flap for mandibular or maxillary reconstruction, it provides significant advantages, particularly for head and neck defects [6,7,8,9,10].

Apart from all these advantages, potential sequelae and impairments following flap harvest must be taken into account as well. Although donor site morbidity is generally considered as being low, it has not been quantified by validated assessment tools so far. Therefore, the main purpose of the study was to evaluate and quantify the donor site morbidity of patients who underwent ablative head and neck surgery followed by reconstruction with a SAFF by applying the Disabilities of the Arm, Shoulder and Hand (DASH) questionnaire.

## 2. Material and Methods

### 2.1. Study Cohort

We performed a multicentric cross-sectional study which included 20 patients with head and neck cancers in whom a SAFF was used for reconstruction. Patients were treated between February 2015 and January 2021 at *(i)* the Department of Otorhinolaryngology, Head and Neck Surgery, Medical University of Vienna, Austria, *(ii)* the Institute of Head and Neck Diseases, Evangelical Hospital, Vienna, Austria, *(iii)* the Shaukat Khanum Memorial Cancer Hospital, Lahore, Pakistan and *(iv)* the Department of Otolaryngology and Maxillofacial Surgery, Zielona Gora, Poland.

### 2.2. Serratus Anterior Free Flap

The SAFF was harvested identically in all participating centers (Supplementary Figure S1) [7,8]. In brief, patients were positioned in a supine position with an abducted arm and mid-axillary lazy-S incisions were used with an axillary extension. In case of a skin paddle requirement, the incision was extended anteriorly and horizontally, not passing the midclavicular line. Elliptical-shaped skin islands were typically located one to two fingerbreadths below the nipple. After retraction of the anterior border of the latissimus dorsi muscle, the neurovascular pedicle can be easily identified as it runs superficially to the serratus slips downwards, where it pierces the slips within the central third. The 7th to 9th slips are typically used for reconstruction. The anterior rib insertion is dissected first, followed by blunt dissection of the slip and division at the posterior scapular insertion. Finally, the thoracodorsal vessels are dissected from distal to proximal.

### 2.3. Complications and Their Definitions

We differentiated between *(i)* flap *(ii)* donor site and *(iii)* recipient site-related complications. Free flap failure was defined as necrosis of the flap without options for salvage surgery [11]. Successful salvage procedures were not assessed as flap failures, but were, of course, counted as flap-related complications. Lacking recipient site vessel situation hampering successful flap perfusion was also counted as flap-related complications, but not as free flap failures. Any incidences related to either donor or recipient sites requiring a return to the operating room were counted as complications. The DASH questionnaire was additionally used for the validated quantification of donor site morbidity, as described in the following. Minor events, such as seroma formation, wound dehiscence or infection, were not accounted for.

### 2.4. DASH Questionnaire

The DASH questionnaire was initially established by the American Academy of Orthopedic Surgeons to evaluate upper limb-related activities, participation, symptoms and disabilities [12], and has been already applied on occasions [13,14,15]. The DASH questionnaire is currently available in more than 50 languages (https://dash.iwh.on.ca/) (accessed on 1 March 2022) and consists of 30 items evaluating daily activities (*n* = 21), symptoms (*n* = 5), participation and impact on daily life (*n* = 3) and confidence in abilities (*n* = 1), and each of those items is rated from 1 (no symptoms) to 5 (most severe symptoms). When applying the appropriate formula {[(sum of *n* responses/*n*) − 1] × 25}, DASH scores may range from 0 (no disability) to 100 (most severe disability). Considering a DASH score of 10.1 as a healthy norm [16], scores between 0–15, 16–45 and 46–100 were classified as “normal or minimal upper-limb disability”, as “mild disability but still able to work” and as “severe upper-limb disability that prevents from working”. DASH questionnaires were handed out in German, English and Polish languages to Austrian, Pakistani and Polish patients, respectively. The English version is provided as Supplementary Material (Supplementary Figure S2).

### 2.5. Statistical Methods

Statistical analysis was performed using the SPSS software (version 27; IBM SPSS Inc., Chicago, IL, USA). Data are indicated as absolute numbers with corresponding percentages in brackets. Mainly descriptive statistics were applied. The chi-square test was used to assess associations between nominal variables. An unpaired Student’s *t*-test or one-way ANOVA was used to compare means of normally distributed variables with two or more than two independent groups. Post hoc comparisons were applied in the case of significant results of one-way ANOVA analysis. Conversely, the Mann–Whitney U test was applied to non-normally distributed variables. All tests were two-sided and *p*-values below 0.05 were considered statistically significant.

## 3. Results

### 3.1. Patient Cohort

A total of 20 patients were analyzed and included 4 females (20.0%) and 16 (80%) males with a mean patient age of 58.2 ± 10.1 years (range: 28.2–75.3 y). Salvage surgeries were performed in the majority of cases (*n* = 14, 70.0%) and tumors most commonly originated from the oral cavity (*n* = 12; 60.0%). Among them, we had 4 T2 (20.0%), 8 T3 (40.0%) and 8 T4 (40.0%) tumors with negative neck nodes in 14 (70.0%) cases. Hence, our cohort mainly consisted of stage IV tumors (*n* = 12; 60.0%), followed by stage III (*n* = 6; 30.0%) and stage II (*n* = 2; 10.0%) diseases (Table 1).

### 3.2. Applications

**Figure 1:**
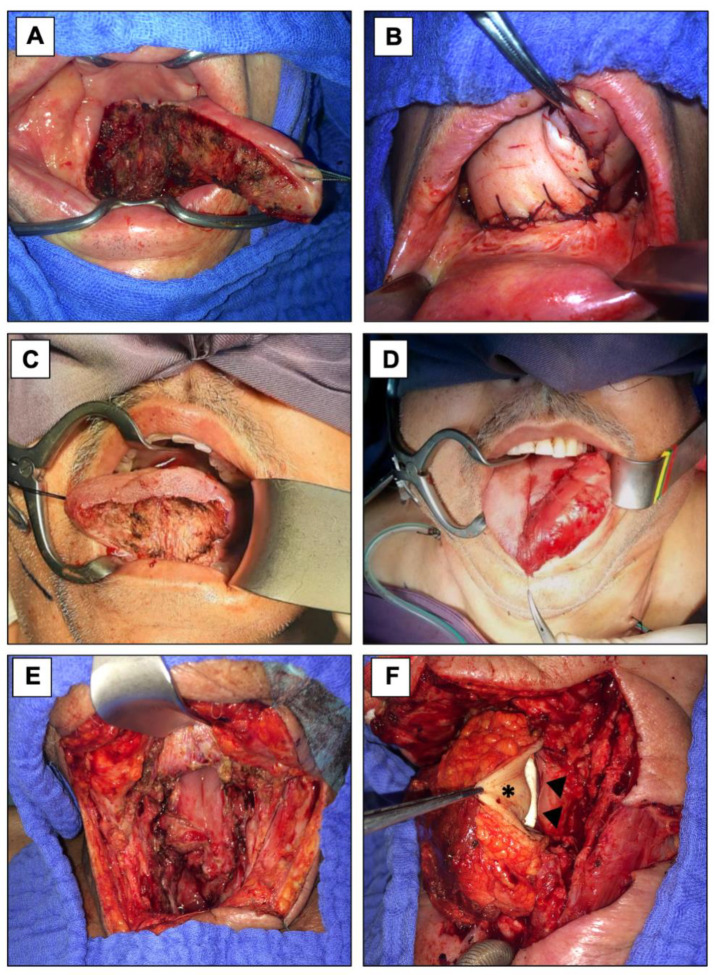
Tongue and Pharyngeal Reconstruction. Tongue reconstruction was performed in two male patients after hemiglossectomy (**A**,**B**) and partial glossectomy (**C**,**D**). Defects were reconstructed with a two-slips myocutaneous SAFF in one case (**A**,**B**) and a one-slip myogenous SAFF (**C**,**D**) in the other. A myocutaneous SAFF was further used as an interposition graft for pharyngeal reconstruction after partial laryngopharyngectomy (**E**). Skin paddle (asterisk) has been tubed over a salivary bypass tube (arrowheads) to reconstruct a partial pharyngeal defect (**F**).

As shown in Table 2, SAFF was most commonly used for tongue reconstruction (*n* = 11; 55.0%) followed by pharyngeal reconstruction after laryngopharyngectomy (*n* = 4; 20.0%; Figure 1). A myocutaneous SAFF was used in 13 cases (65.0%) and mostly for tongue (*n* = 7), pharyngeal (*n* = 4) and skin reconstruction (*n* = 2). The postoperative course was uneventful in 14 patients (70%), while complications were noticed in 6 cases.

### 3.3. Complications and Free Flap Failure

Free flap, recipient site and donor site-related complications were noticed in 4, 1 and 1 cases, respectively (Table 3). In two (SAFF2, 12) of four patients, SAFF was lost intraoperatively due to the absence of sufficient arterial vessels after former chemoradiation and both patients were salvaged with a pectoralis major muscle flap (PMMF). The other two male patients with stage IV hypopharyngeal tumors experienced vascular occlusion on POD 3 and 9 (SAFF13, 15) and ended up with flap loss and pharyngocutaneous formation (PCF). Hence, we had a free flap survival rate of 88.9% (16/18), excluding those two cases with intraoperative flap loss due to missing recipient vessels.

In addition, PCF formation as a sole recipient site complication was noticed in one patient (SAFF16) after salvage laryngectomy and reconstruction with myogenous SAFF. Similarly, donor site-related problems with revision surgery occurred also in only one patient (SAFF5), who showed up with subacute bleeding and hematoma formation. Altogether, complemental PMMF was applied in five cases, including two patients with intraoperative flap loss, two with delayed flap loss after vascular occlusion, and one patient, to patch persistent PCF formation.

### 3.4. Donor Site Morbidity

**Figure 2:**
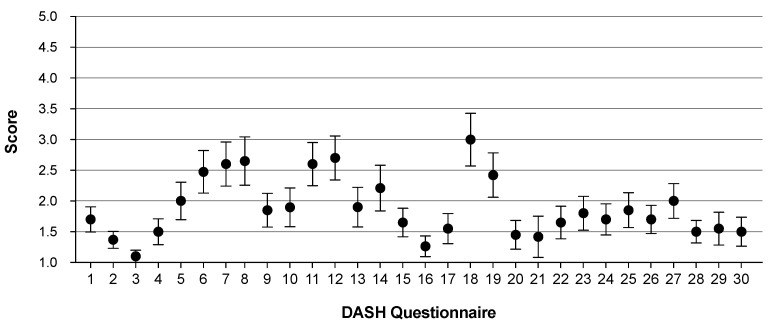
DASH questionnaire. Ratings (Score 1–5) are illustrated as mean ± standard error of the mean (SEM) for all questions.

Donor site morbidity was evaluated with a mean (median) time of 6.4 (4.0) months after surgery (range: 3.0–18.0 months) using the DASH questionnaire. In detail, 40% of patients (*n* = 8) indicated normal or minimal upper-limb disability (DASH score 0–15), 50% (*n* = 10) indicated mild disabilities but with a preserved ability to work (DASH score 16–45), while 10% (*n* = 2) indicated even severe upper-limb disability that prevents them from working (DASH score 46–100). We detected three times higher DASH scores in patients with free flap failure (46.0 vs. 15.5; *p* = 0.039), while the DASH score was neither affected by sex (*p* = 0.303), age (*p* = 0.872) nor salvage procedures (*p* = 0.378). Similarly, in the case where an additional PMMF harvest was necessary, DASH scores were 2.6-times higher compared to those with uneventful postoperative courses (39.9 vs. 15.47; *p* = 0.081). Notably, the harvest of myocutaneous SAFF was also associated with higher, but not significantly different DASH scores (26.1 vs. 13.1; *p* = 0.159). Regarding the DASH questionnaire, in particular, the highest average ratings indicating mild to moderate difficulties were given to questions # 18 (recreational activities in which you take some force or impact through your arm, shoulder or hand), # 12 (change a light bulb overhead) and # 8 (garden or do yard work); (Figure 2; Table 4).

## 4. Discussion

Due to its thin, finger-shaped fashion and reliable vascular supply, the SAFF provides enormous flexibility to restore any defect in the head and neck region. It has therefore found many potential applications in the head and neck, ranging from covering anterior skull base defects [17] over dynamic facial reanimation [18,19] to tongue reconstruction [8,20].

Our study confirms the versatility of the SAFF for head and neck reconstruction [6,21]. When it comes to the reconstruction of the tongue, the SAFF affords an adequate amount of bulk to restore any defects ranging from partial to total glossectomies with satisfying functional outcomes, either with or without skin paddles [8,20]. The excellent epithelialization of the myogenous SAFF was already mentioned before [10] and proven by our study group.

Despite these unquestionable benefits and various applications, any free flap use must always be balanced with disadvantages or drawbacks. Therefore, we set up this study to prove the flexibility of the SAFF for head and neck reconstruction alongside with low to almost absent donor site morbidity. Considering literature, scapular winging was occasionally reported [22], while the overwhelming majority of data indicates a neglectable donor site morbidity without significant shoulder dysfunction [10,23,24]. It is important to emphasize that the lowest three to four digitations are typically harvested, allowing the remaining slips to stabilize the scapular to prevent scapular winging [19]. Even the additional harvest of the long thoracic nerve for dynamic facial reanimation is not associated with higher donor site morbidity [18,19,25,26]. Beyond that, hematoma and seroma formation are described as the most common complications occurring in 6% to 10% of cases [22,23,27]. Similarly, hematoma formation with a need for surgical revision was necessary for one of our patients (5%).

However, objective evaluation of donor site morbidity after SAFF harvest is still lacking. We are, to the best of our current knowledge, the first group who objectively evaluated donor site morbidity by using the DASH questionnaire. According to Hunsaker et al. (1992), a DASH score of 10.1 is considered as a reference value for a healthy cohort [16]. A mean DASH score of 20 (normal 0–15) in our cohort indicates an almost normal upper limb function with a minimal to a mild disability, but the preserved capability to work normally. In more detail, the greatest impairments that were largely rated as moderate were reported during exhausting activities, such as gardening or recreational activities requiring some force of impact through the upper limb. Similar to previous works [22,23,24], all of our patients were, however, able to maintain their normal daily lives without significant restrictions.

In comparison, the RFFF and the ALT flap represent the workhorse flaps for head and neck reconstruction so far, which, of course, carry also morbidities at the donor site. Wound healing complications, split-thickness graft necrosis and exposure of the tendon are reported in 10–15% of cases with a decreased, measurable dexterity and function after RFFF harvest [28,29,30]. Similarly, a harvest of an ALT flap significantly affects leg extension and flexion, and lateral thigh paresthesia is reported most frequently (24.0%) followed by musculoskeletal dysfunction (4.8%) or wound dehiscence (4.8%) [31,32]. However, overall, patients are usually not affected in their daily lives, which is true also for patients after the SAFF harvest.

Although all flaps proved to have low donor site morbidity, they offer completely different characteristics for head and neck reconstruction. For instance, the thin and pliable RFFF is excellent for mucosal or skin reconstruction if no additional soft tissue is required. Completely opposite to this, the ALT flap provides large skin paddles and extensive volume for large soft tissue defects. In turn, the SAFF offers less volume than the ALT but more than the RFFF, which can be easily modified due to the centrally entering vascular pedicle with its reliable anatomy. Therefore, we see the role of the SAFF between the RFFF and the ALT, with particular advantages for tongue and facial reconstruction.

Notably, patients who additionally required PMMF harvest showed almost three times higher DASH scores. The PMMF, as a robust and easy to harvest pedicled flap, generally represents the workhorse backup flap for head and neck reconstruction. Particularly, the PMMF is used as a backup flap if *(i)* the free flap fails, *(ii)* elderly patients will not tolerate long operation times or *(iii)* patients with vessel-depleted necks need a reconstruction of the defects [33]. Nonetheless, the PMMF also carries disadvantages, such as significant donor site morbidity with arm and shoulder dysfunction, which has also been true for our cohort [33,34,35]. Altogether, our data indicate that PMMF harvest significantly adds or even multiplies donor site morbidity of the SAFF, which needs stronger consideration in salvage settings.

We applied the PMMF in salvage situations to either repair free flap failures (*n* = 4) or to cover PCF (*n* = 1) formation after salvage laryngectomy. Excluding those two patients with vessel-depleted necks due to previous chemoradiation, we had a free flap survival rate of 88.9%. This is lower when compared to the literature reporting free flap survival rates of 95–97% after head and neck reconstructions and salvage rates of 20–30% [36,37,38]. In comparison, 70% of our patients were in salvage settings, which may represent an explanation for the lower free flap survival rate, as previous radiotherapy represents a known risk factor for significantly higher free flap failure rates [39].

## 5. Conclusions

Using the SAFF for head and neck reconstruction is associated with low to moderate donor site morbidity, and patients are hardly affected by the harvest. Harvest of PMMF as a backup flap in salvage situations significantly multiplies donor site morbidity and subsequently upper limb disability, which needs to be strongly considered in the future.

## Figures and Tables

**Table 1:** Patient Cohort and DASH Score.

		DASH Score	
VARIABLES	*n* (%)	*Mean (Median) ± SEM (SD)*	*p*-Value
**Sex**			
F	4 (20.0)	30.8 (23.2) ± 14.6 (29.2)	
M	16 (80.0)	19.3 (19.9) ± 4.2 (16.9)	0.303 ^a^
**Age**			
<56	10 (50.0)	20.8 (14.0) ± 6.8 (21.4)	
≥56	10 (50.0)	22.3 (20.0) ± 5.9 (18.7)	0.872 ^a^
**Tumor Site**			
Oral Cavity	12 (60.0)	16.6 (13.0) ± 4.0 (13.9)	
Larynx	2 (10.0)	10.4 (10.4) ± 5.4 (7.6)	
Hypopharynx	4 (20.0)	42.3 (42.7) ± 15.3 (30.7)	
Other	2 (10.0)	20.9 (20.9) ± 0.9 (1.2)	0.106 ^b^
**T-Classification**			
T1	0 (0.0)	-	
T2	4 (20.0)	18.9 (19.9) ± 5.2 (10.4)	
T3	8 (40.0)	18.0 (10.9) ± 7.5 (21.2)	
T4	8 (40.0)	26.4 (20.9) ± 7.9 (22.3)	0.683 ^b^
**N-Classification**			
N0	14 (70.0)	22.0 (20.0) ± 4.9 (18.3)	
N1	2 (10.0)	9.1 (9.1) ± 3.0 (4.3)	
N2	4 (20.0)	26.2 (17.9) ± 14.5 (29.0)	
N3	0 (0.0)	-	0.618 ^b^
**Tumor Stage**			
Stage I	0 (0.0)	-	
Stage II	2 (10.0)	19.9 (19.9) ± 0.1 (0.1)	
Stage III	6 (30.0)	12.5 (10.9) ± 4.1 (10.1)	
Stage IV	12 (60.0)	26.4 (20.9) ± 6.7 (23.3)	0.386 ^b^
**Salvage Situation**			
Yes	14 (70.0)	24.2 (19.9) ± 5.9 (22.3)	
No	6 (30.0)	15.5 (16.1) ± 4.2 (10.2)	0.378 ^a^
**Skin Paddle**			
Yes	13 (65.0)	26.1 (20.0) ± 6.1 (21.8)	
No	7 (35.0)	13.1 (6.0) ± 4.3 (11.4)	0.159 ^a^

**Abbreviations: SEM**, standard error of the mean; **SD**, standard deviation; **F**, female; **M**, male; ^a^ Unpaired Student’s *t*-test; ^b^ One-Way ANOVA.

**Table 2:** Applications.

Case	Application	Sex	Age	Skin Paddle	Salvage Setting	Stage	Complication	DASH	Assessment
1	Tongue Reconstruction	M	58.8	Yes	Yes	IV	No	40.70	4
2		M	28.2	No	Yes	IV	Yes	34.50	3
3		F	52.7	Yes	No	IV	No	30.56	3
4		M	51.1	Yes	Yes	III	No	26.70	3
5		M	65.5	No	No	III	Yes	20.75	3
6		M	62.2	Yes	Yes	II	No	20.00	15
7		M	54.9	No	No	III	No	6.03	6
8		F	66.9	Yes	Yes	IV	No	5.20	7
9		M	50.3	Yes	Yes	III	No	5.00	4
10		M	66.4	No	No	IV	No	3.50	3
11		M	55.3	Yes	Yes	III	No	0.90	6
		**9 (81.8)**	**55.6**	**7 (63.6)**	**7 (63.6)**	**7 (63.6)**	**2 (18.2)**	**17.6**	**5.2**
12	Pharyngeal Reconstruction	F	54.3	Yes	Yes	IV	Yes	71.7	13
13		M	75.3	Yes	Yes	IV	Yes	65.5	3
14		M	64.9	Yes	Yes	II	No	19.8	4
15		M	55.6	Yes	No	IV	Yes	12.1	18
		**3 (75.0)**	**62.5**	**4 (100.0)**	**3 (75.0)**	**3 (75.0)**	**3 (75.0)**	**42.3**	**9.5**
16	Overlay Technique	F	53.0	No	Yes	III	Yes	15.8	13
17		M	54.7	No	Yes	IV	No	5.0	8
		**1 (50.0)**	**53.8**	**0 (0.0)**	**2 (100.0)**	**1 (50.0)**	**1 (50.0)**	**10.4**	**10.5**
18	Skin Reconstruction	M	61.1	Yes	Yes	IV	No	21.7	5
19		M	74.1	Yes	No	IV	No	20.0	4
		**2 (100.0)**	**67.6**	**2 (100.0)**	**1 (50.0)**	**2 (100.0)**	**0 (0.0)**	**20.8**	**4.5**
20	Maxillary Reconstruction	M	58.8	No	Yes	IV	No	5.83	3
		**1 (100.0)**	**58.8**	**0 (0.0)**	**1 (100.0)**	**1 (100.0)**	**0 (0.0)**	**5.83**	**3**

Sociodemographic characteristics and DASH scores are shown according to application. The ratio of males, myocutaneous flaps, salvage settings, stage IV disease and complications are indicated as well as the mean age (years) and the mean DASH scores.

**Table 3:** Complications.

Case	Complication	Type	Salvage Procedure
SAFF 2	Intraoperative flap loss	Flap related	PMMF
SAFF 5	Subacute bleeding at donor site	Donor site related	Revision Surgery
SAFF 12	Intraoperative flap loss	Flap related	PMMF
SAFF 13	Vascular occlusion on POD 3 with delayed flap loss and PCF formation	Flap related	PMMF
SAFF 15	Vascular occlusion on POD 9 with delayed flap loss and PCF formation	Flap related	PMMF
SAFF 16	Persistent PCF	Recipient site related	PMMF

**Abbreviations: SAFF**, serratus anterior free flap; **PMMF**, pectoralis major muscle flap; **POD**, postoperative day; **PCF**, pharyngocutaneous fistula.

**Table 4:** DASH questionnaire.

Questions		Mean	Median	SD
**1**	Open a tight or new jar	1.7	1	0.92
**2**	Write	1.4	1	0.60
**3**	Turn a key	1.1	1	0.45
**4**	Prepare a meal	1.5	1	0.95
**5**	Push open a heavy door	2.0	1	1.33
**6**	Place an object on a shelf above your head	2.5	2	1.50
**7**	Do heavy household jobs	2.6	2	1.60
**8**	Garden or outdoor property work	2.7	2.5	1.76
**9**	Make a bed	1.9	1	1.22
**10**	Carry a shopping bag or briefcase	1.9	1	1.41
**11**	Carry a heavy object (over 5kgs)	2.6	2.5	1.57
**12**	Change a lightbulb overhead	2.7	2	1.59
**13**	Wash or blow dry your hair	1.9	1	1.45
**14**	Wash your back	2.2	1	1.62
**15**	Put on a jumper	1.7	1	1.04
**16**	Use a knife to cut food	1.3	1	0.73
**17**	Recreational activities which require little effort	1.6	1	1.10
**18**	Recreational activities with require you to take some force or impact through your arm, should or hand	3.0	2.5	1.81
**19**	Recreational activities in which you move your arm freely	2.4	2	1.57
**20**	Manage transport needs	1.5	1	1.05
**21**	Sexual activities	1.4	1	1.16
**22**	During the past week, to that extent has your arm, shoulder or hand problem interfered with your normal social activities with family, friends, neighbors or groups?	1.7	1	1.18
**23**	During the past week, were you limited in your work or other regular daily activities as a result of your arm, shoulder or hand problem?	1.8	1	1.24
**24**	Arm, shoulder or hand pain	1.7	1	1.13
**25**	Arm, shoulder or hand pain when you do any specific activity	1.9	1	1.27
**26**	Tingling (pins and needles) in your arm, shoulder or hand	1.7	1	1.03
**27**	Weakness in your arm, shoulder or hand	2.0	1.5	1.26
**28**	Stiffness in your arm, shoulder or hand	1.5	1	0.83
**29**	During the past week, how much difficulty have you had sleeping because of the pain in your arm, shoulder or hand	1.6	1	1.19
**30**	I feel less capable, less confident or less useful because of my arm, shoulder or hand problem	1.5	1	1.05
**Total**		**21.3**	**19.9**	**19.6**

DASH questionnaires are indicated with the mean and median DASH scores of our cohort. Standard deviations (SD) are given additionally.

## Data Availability

Data are available on reasonable request.

## Supplementary Materials

The following supporting information can be downloaded at: https://www.mdpi.com/article/10.3390/jcm11092397/s1, Figure S1: Harvest of SAFF; Figure S2: DASH Questionnaire (English Version).
