# Supplementary material for: Using the DASH Questionnaire to Evaluate Donor Site Morbidity of the Serratus Anterior Free Flap in Head and Neck Reconstruction: A Multicenter Study
Source: J Clin Med. 2022 Apr 25;11(9):2397. doi: 10.3390/jcm11092397 (PMC9101023; doi:10.3390/jcm11092397)
Supplement: Supplementary file 1 [file jcm-11-02397-s001.zip › jcm-1672341-Figure S2.pdf]

DISABILITIES OF THE ARM, SHOULDER AND HAND – British English

THE DASH

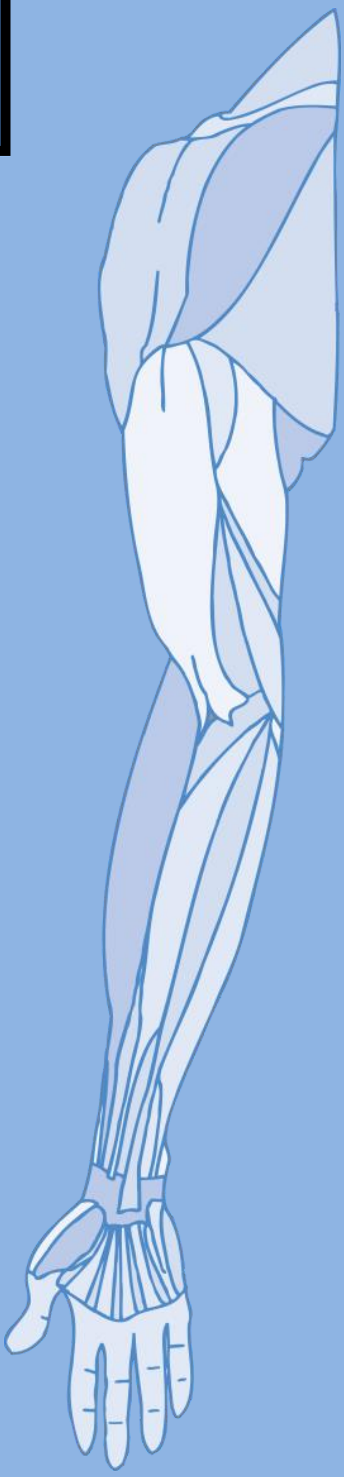

INSTRUCTIONS

This questionnaire asks about your symptoms as well as your ability to do certain activities.

Please answer *every question*, based on your condition in the last week, by circling the appropriate number.

If you did not do an activity in the last week, please give your *best guess* which response would be the most accurate.

It doesn't matter which hand or arm you use to do the activity; please answer based on your ability regardless of how you do the task.

© Institute for Work & Health 2006. All rights reserved.

British English translation courtesy of:
Prof Alison Hammond[1], Dr Yeliz Prior[1], Prof Sarah Tyson[2]
[1] Centre for Health Sciences Research, University of Salford;
[2] Centre for Long Term Conditions Research, University of Manchester, UK.

DISABILITIES OF THE ARM, SHOULDER AND HAND – British English

Please rate your ability to do the following activities in the last week by circling the number below the appropriate response.

	NO DIFFICULTY	MILD DIFFICULTY	MODERATE DIFFICULTY	SEVERE DIFFICULTY	UNABLE
1. Open a tight or new jar	1	2	3	4	5
2. Write	1	2	3	4	5
3. Turn a key	1	2	3	4	5
4. Prepare a meal.	1	2	3	4	5
5. Push open a heavy door	1	2	3	4	5
6. Place an object on a shelf above your head	1	2	3	4	5
7. Do heavy household jobs (e.g. wash windows, clean floors)	1	2	3	4	5
8. Garden or outdoor property work	1	2	3	4	5
9. Make a bed	1	2	3	4	5
10. Carry a shopping bag or briefcase	1	2	3	4	5
11. Carry a heavy object (over 10 lbs/ 5kgs)	1	2	3	4	5
12. Change a lightbulb overhead	1	2	3	4	5
13. Wash or blow dry your hair	1	2	3	4	5
14. Wash your back	1	2	3	4	5
15. Put on a jumper	1	2	3	4	5
16. Use a knife to cut food	1	2	3	4	5
17. Recreational activities which require little effort (e.g. card playing, knitting, etc)	1	2	3	4	5
18. Recreational activities which require you to take some force or impact through your arm, shoulder or hand (e.g. golf, hammering, tennis etc)	1	2	3	4	5
19. Recreational activities in which you move your arm freely (e.g. playing Frisbee, badminton etc)	1	2	3	4	5
20. Manage transport needs (getting from one place to another)	1	2	3	4	5
21 Sexual activities	1	2	3	4	5

	NOT AT ALL	SLIGHTLY	MODERATELY	QUITE A BIT	EXTREMELY
22. During the past week, *to what extent* has your arm, shoulder or hand problem interfered with your normal social activities with family. friends, neighbours or groups? *(circle number)*	1	2	3	4	5

	NOT LIMITED AT ALL	SLIGHTLY LIMITED	MODERATELY LIMITED	VERY LIMITED	UNABLE
23. During the past week, were you limited in your work or other regular daily activities as a result of your arm, shoulder or hand problem? *(circle number)*	1	2	3	4	5

**Please rate the severity of the following symptoms in the last week** *(circle number)*	NONE	MILD	MODERATE	SEVERE	EXTREME
24. Arm, shoulder or hand pain	1	2	3	4	5
25. Arm, shoulder or hand pain when you do any specific activity	1	2	3	4	5
26. Tingling (pins and needles) in your arm, shoulder or hand	1	2	3	4	5
27. Weakness in your arm, shoulder or hand	1	2	3	4	5
28. Stiffness in your arm, shoulder or hand	1	2	3	4	5

	NO DIFFICULTY	MILD DIFFICULTY	MODERATE DIFFCULTY	SEVERE DIFFICULTY	SO MUCH DIFFICULTY THAT I CAN'T SLEEP
29 During the past week, how much difficulty have you had sleeping because of the pain in your arm, shoulder or hand? *(circle number)*	1	2	3	4	5

	STRONGLY DISAGREE	DISAGREE	NEITHER AGREE OR DISAGREE	AGREE	STRONGLY AGREE
30. I feel less capable, less confident or less useful because of my arm, shoulder or hand problem *(circle number)*	1	2	3	4	5

DASH DISABILITY/SYMPTOM SCORE = [(sum of n responses) –1] x 25 (where n is the number of completed responses)
n

A DASH score may not be calculated if there are greater than 3 missing items.

WORK MODULE (OPTIONAL)

The following questions ask about the impact of your arm, shoulder or hand problem on your ability to work (**including** home-making, if that is your main work role).

Please indicate what your job / work is: ____

❑ I do not work (you may skip this section).

Please circle the number that best describes your physical ability in the past week. Did you have any difficulty:

	NO DIFFICULTY	*MILD DIFFICULTY*	*MODERATE DIFFICULTY*	*SEVERE DIFFICULTY*	*UNABLE*
1. Doing your work in your usual way?	1	2	3	4	5
2. Doing your usual work because of arm, shoulder or hand pain?	1	2	3	4	5
3. Doing your work as well as you would like?	1	2	3	4	5
4. Spending your usual amount of time doing your work?	1	2	3	4	5

SPORTS/PERFORMING ARTS MODULE (OPTIONAL)

The following questions relate to the impact of your arm, shoulder or hand problem on playing either *your musical instrument* or *sport* or *both*. If you play more than one sport or instrument (or play both), please answer with respect to that activity which is most important to you.

Please indicate the sport or instrument which is most important to you: __________________________________

❑ I do not play a sport or an instrument. (You may skip this section).

Please circle the number that best describes your physical ability in the past week. Did you have any difficulty:

	NO DIFFICULTY	*MILD DIFFICULTY*	*MODERATE DIFFICULTY*	*SEVERE DIFFICULTY*	*UNABLE*
1. Playing your instrument or sport in your usual way?	1	2	3	4	5
2. Playing your musical instrument or sport because of arm, shoulder or hand pain?	1	2	3	4	5
3. Playing your instrument or sport as well as you would like?	1	2	3	4	5
4. Spending your usual amount of time practising or playing your instrument or sport?	1	2	3	4	5

Scoring the optional modules: add up the assigned values for each response;
Divide by 4 (number of items); subtract 1; multiple by 25.
An optional module score may not be calculated if there are any missing items.
