# Supplementary figures and images for: Using the DASH Questionnaire to Evaluate Donor Site Morbidity of the Serratus Anterior Free Flap in Head and Neck Reconstruction: A Multicenter Study
Source: J Clin Med. 2022 Apr 25;11(9):2397. doi: 10.3390/jcm11092397 (PMC9101023; doi:10.3390/jcm11092397)

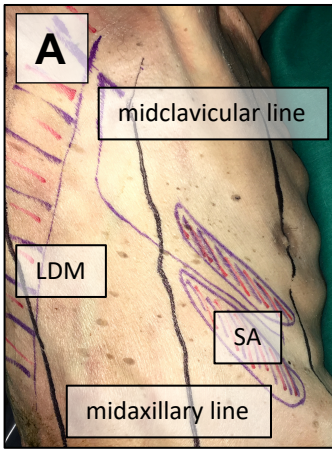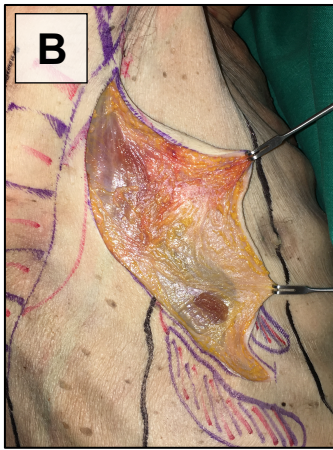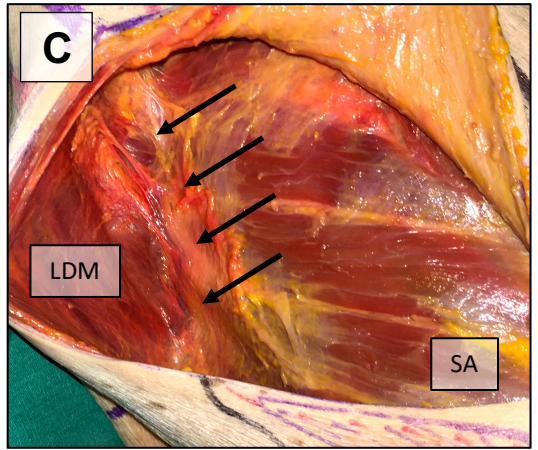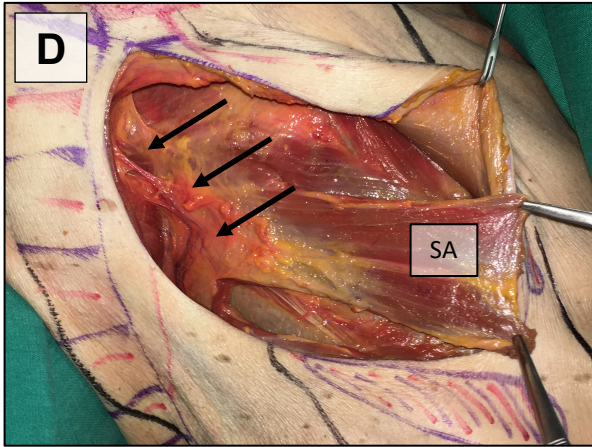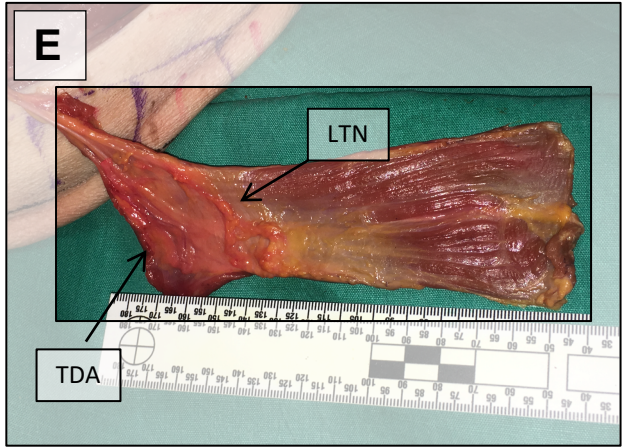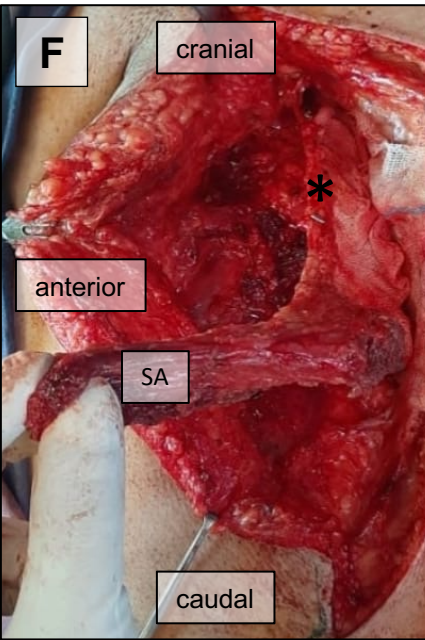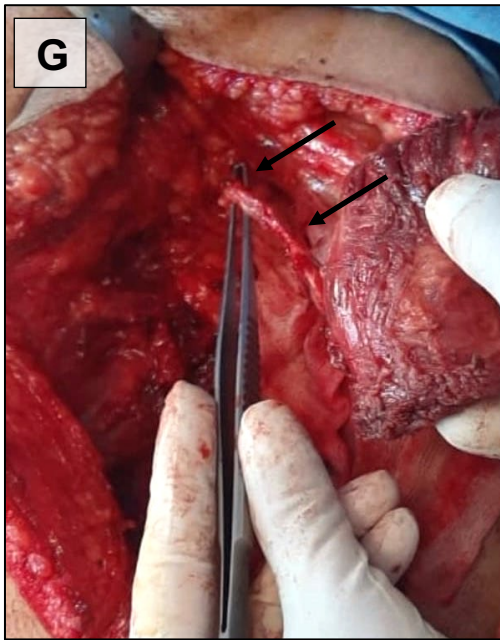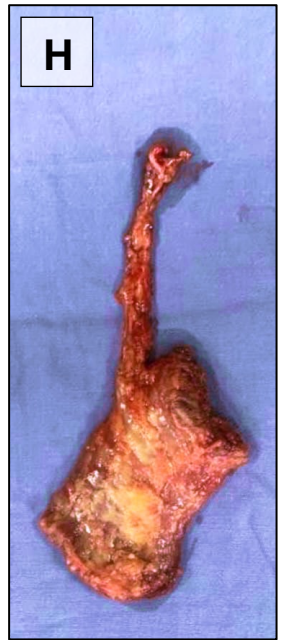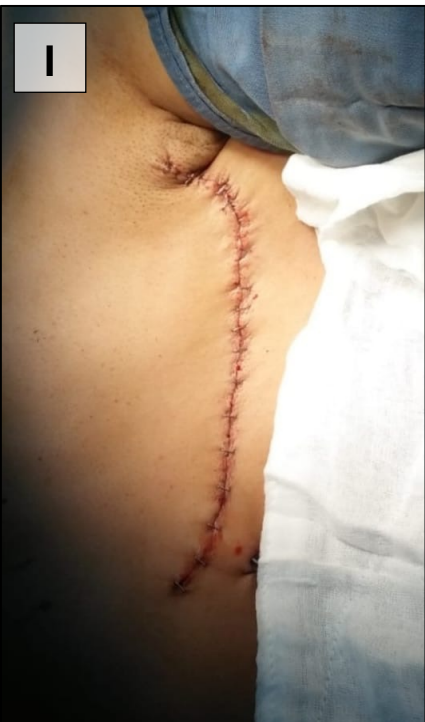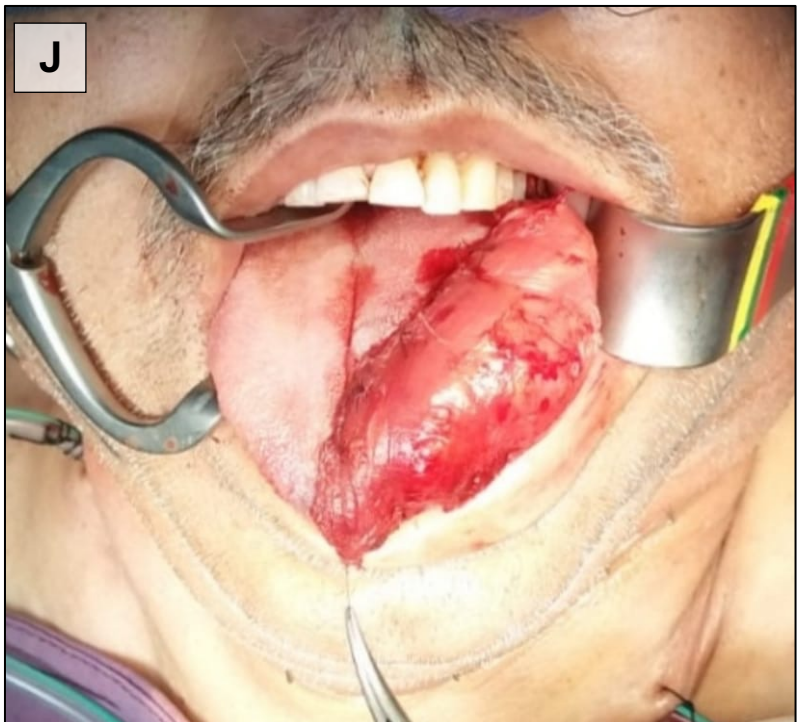

Supplement: Supplementary file 1 [file jcm-11-02397-s001.zip › jcm-1672341-Figure S1.pdf]
